# A Hybrid Deep Learning Method for Early and Late Mild Cognitive Impairment Diagnosis With Incomplete Multimodal Data

**DOI:** 10.3389/fninf.2022.843566

**Published:** 2022-03-15

**Authors:** Leiming Jin, Kun Zhao, Yan Zhao, Tongtong Che, Shuyu Li

**Affiliations:** ^1^Beijing Advanced Innovation Center for Biomedical Engineering, School of Biological Science and Medical Engineering, Beihang University, Beijing, China; ^2^State Key Lab of Cognition Neuroscience and Learning, Beijing Normal University, Beijing, China

**Keywords:** MCI, GAN, classification, incomplete data, multimodality

## Abstract

Multimodality neuroimages have been widely applied to diagnose mild cognitive impairment (MCI). However, the missing data problem is unavoidable. Most previously developed methods first train a generative adversarial network (GAN) to synthesize missing data and then train a classification network with the completed data. These methods independently train two networks with no information communication. Thus, the resulting GAN cannot focus on the crucial regions that are helpful for classification. To overcome this issue, we propose a hybrid deep learning method. First, a classification network is pretrained with paired MRI and PET images. Afterward, we use the pretrained classification network to guide a GAN by focusing on the features that are helpful for classification. Finally, we synthesize the missing PET images and use them with real MR images to fine-tune the classification model to make it better adapt to the synthesized images. We evaluate our proposed method on the ADNI dataset, and the results show that our method improves the accuracies obtained on the validation and testing sets by 3.84 and 5.82%, respectively. Moreover, our method increases the accuracies for the validation and testing sets by 7.7 and 9.09%, respectively, when we synthesize the missing PET images *via* our method. An ablation experiment shows that the last two stages are essential for our method. We also compare our method with other state-of-the-art methods, and our method achieves better classification performance.

## Introduction

Alzheimer’s disease (AD) is one of the most common irreversible neurodegenerative diseases with progressive and irreversible characteristics, and sufferers of AD account for 50–80% (Guillon et al., 2017; Zhang et al., 2018; Alzheimer’s Association, 2019) of all types of dementia patients. Mild cognitive impairment (MCI), which is a transitional stage between normal aging and AD, can be subdivided into early MCI (EMCI) and late MCI (LMCI) according to the extent of episodic memory impairment (Aisen et al., 2010). Previous studies have confirmed that LMCI exhibits a higher risk of converting to AD than EMCI (Jessen et al., 2014). Thus, the accurate classification of EMCI and LMCI is crucial for the precise treatment of MCI patients.

Recently, neuroimaging data have been widely used in the early diagnosis of AD (Ye et al., 2016). Early studies concentrated on single-modality data such as magnetic resonance imaging (MRI) or positron emission tomography (PET) images (Higdon et al., 2004; McEvoy et al., 2009). However, these studies ignored the inherently complementary information of different modalities, which could reduce the accuracy of early detection. Thus, increasingly many studies have used multimodal data for the early diagnosis of AD and have shown that the proper use of different modalities of data can improve the accuracy of disease diagnosis (Zhang et al., 2011; Gray et al., 2013; Liu et al., 2018a; Zhou et al., 2019). Zhou et al. (2019) presented a novel three-stage deep feature learning and fusion framework to make full use of data with different modalities, and the results indicated that the multimodal method yielded a statistically significant improvement over the single-modality method.

However, the missing data problem is a crucial challenge when using multimodal data (Marlin, 2008; Liu et al., 2017). For example, many subjects have been scanned by MRI, while only a few have PET scans because of their high cost or radioactive exposure. Previous methods only used subjects with paired MRI and PET images (Zhang and Shen, 2012). However, such a crude strategy affects the performance of the resulting model due to the decrease in number of training subjects. To make full use of all available data, a more appropriate approach is to impute instead of discarding the missing PET data. Previous studies imputed missing features instead of images (Marlin, 2008; Abdelaziz et al., 2021). However, these imputed features are defined by experts according to their different types of prior knowledge and may not be discriminative for early AD diagnosis. Recently, generative adversarial networks (GANs) have been used to impute missing data by image synthesis (Wolterink et al., 2017; Pan et al., 2018; Yang et al., 2018; Zhao et al., 2021). These networks used the underlying relevance between MRI and PET images to synthesize missing PET scans.

In this study, we focused on the classification of EMCI and LMCI using multimodal images with PET image imputation. However, traditional methods simply generate images and use them for early AD diagnosis. Such studies independently train the two networks without any information communication (Pan et al., 2018; Zhao et al., 2021). Gao et al. (2021) proposed a deep learning framework to integrate a task-induced pyramid and attention GAN (TPA-GAN) that designed a task-induced discriminator to simultaneously learn image generation and disease classification. However, this approach must design an additional discriminator network for classification, which may increase the computational burden. Pan et al. (2019, 2021) proposed a disease-image-specific deep learning (DSDL) framework for joint neuroimage synthesis and disease diagnosis. However, this method encourages feature maps of a synthetic image to be consistent with those of its corresponding real image instead of fusion features of MRI and PET images. Here, the main goal is to classify EMCI and LMCI. Thus, the GAN must focus on the crucial brain regions that are helpful for disease classification instead of the overall image. Additionally, the classification network should be suitable for these synthesized images while considering the differences between the synthesized images and the real images.

Specifically, we propose a hybrid deep learning method, i.e., a three-stage deep learning framework, to classify EMCI and LMCI with incomplete multimodal datasets. In the first stage, the classification network is pretrained *via* paired MRI and PET images. In the second stage, our goal is to train a GAN for image synthesis. We regard the pretrained classification network without the final fully connected (FC) layer as a fusion model that can extract and fuse the features of different data modalities. We freeze the parameters of the fusion model and compare the differences between the fused features of real MRI and real PET images and those of real MRI and synthesized PET images. By introducing the differences as a loss into the GAN, the generator can better focus on the helpful regions for classification. In the last stage, we aim to fine-tune the classification network to better adapt to the synthesized images. We assume that the PET images in the training set are missing. We freeze the parameters of the generator trained in the second stage and use it to synthesize PET images. Afterward, real MR images and generated PET images are used to fine-tune the classification model, which has been pretrained in the first stage. Thus, the classification network can better adapt to the synthesized images, which is of great significance for solving the problem of missing data. Finally, we iterate stages 2 and 3 several times to obtain the best models.

In summary, the main contributions of our work are as follows. (1) We integrate a classification network into a GAN to generate images that are helpful for classification; (2) we use the synthesized images to fine-tune the classification model so that it can adapt to synthesized images; (3) we iteratively train the classification network and GAN to improve the performance of the two networks.

## Materials and Methods

**FIGURE 1:**
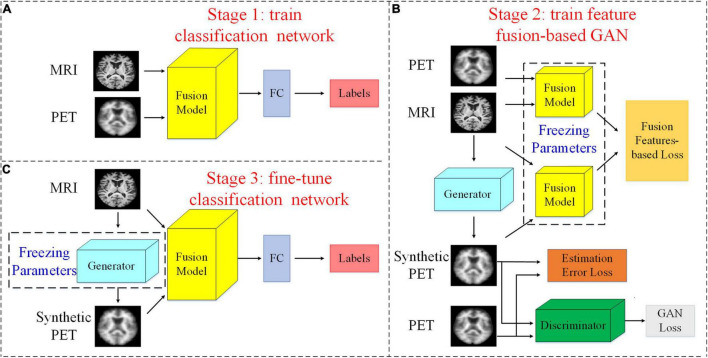
Flowchart of our study. **(A)** Stage 1: We pretrain the classification network with both MRI and PET images and their corresponding labels. **(B)** Stage 2: The feature fusion-based GAN is trained using the fusion model that is composed of all layers except the FC layer of the classification network. **(C)** stage 3: We use the real MR images, synthesized PET images, and their corresponding labels to fine-tune the classification model. Here, we assume that the PET images are missing and use the generator to synthesize them.

In this section, we introduce our proposed method. An overview of our study can be found in Figure 1. The flowchart shows that our study consists of three stages: (1) a pretraining classification network; (2) a feature fusion-based training GAN (FF-GAN); (3) fine-tuning of the classification model. In the following section, we explain the details of each stage.

### Pretraining Classification Network

We use paired MRI and PET images to pretrain a classification network that can be used for the early diagnosis of AD. As shown in Figure 1A, for this classification network, we regard all layers except the last FC layer as a feature fusion model. After the last FC layer, we can obtain the classification results. Therefore, we regard the FC layer as a classifier. Thus, the fusion model can extract and fuse data with different modalities and obtain the features that are directly used in the classifier. Thus, these features are most helpful for classification. In the next stage, we freeze the parameters of the fusion model and use it to design a new loss function to make the GAN better able to generate helpful images for classification.

### Feature Fusion-Based Generative Adversarial Network

#### Generative Adversarial Network

Goodfellow et al. (2014) first introduced the GAN (Goodfellow et al., 2014), the goal of which is to minimize the difference between a generated distribution and the real distribution. Our method is to improve on the traditional GAN. Thus, we use the original minimax game, which can be expressed as:

$$\arg\,\min_{G}\max_{D}L_{G\,A\,N}\,\left(G,D\right)=$$

(1) $$E_{x∼P_{d\,a\,t\,a}}\left[\log D\left(x\right)\right]+E_{z∼P_{n\,o\,i\,s\,e\,\left(z\right)}}[\log\left(1-D\left(G\left(z\right)\right)\right].$$

In this study, we use real 3D MRI patches to replace random noise as the input. Specifically, the generator network G generates 3D PET patches by transforming the data distribution obtained from real MRI results. Thus, the objective function can be rewritten as:

$$\arg\,\min_{G}\max_{D}L_{G\,A\,N}\,\left(G,D\right)=$$

(2) $$E_{x∼P_{P\,E\,T}}\left[\log D\left(x\right)\right]+E_{z∼P_{M\,R\,I}}[\log\left(1-D\left(G\left(z\right)\right)\right].$$

To make the synthetic PET images and the real images more similar, we employ the L1 norm estimation error to reduce blurring, and the loss function is introduced only into the generator’s task (Wang et al., 2018). The L1 loss function is as follows:


(3) $$L_{1}\,\left(G\right)=E_{\text{y}∼P_{P\,E\,T},x∼P_{M\,R\,I}}\,\left[{||y-G\,\left(x\right)||}_{1}\right].$$

The final loss function of our baseline GAN can be expressed as:


(4) $$\arg\,\min_{G}\max_{D}L_{G\,A\,N}\,\left(G,D\right)+\lambda_{1}\,L_{1}\,\left(G\right).$$

#### Feature Fusion-Based Loss

Traditional methods usually design additional loss functions according to the difference between the generated image and the real image. For example, Yang et al. (2018) applied a perceptual loss function to retain generated image details and information. They adopted a pretrained Visual Geometry Group (VGG)-19 network to extract features from both real images and synthesized images. The differences between these features were used to construct a loss function. However, the VGG-19 network was pretrained with natural images. Therefore, it might not extract the features of medical images effectively. In addition, this method ensures the similarity of the features of the generated images and real images, but in fact, we fuse MRI and PET data for classification instead of single-modality data. We should guarantee the consistency of fused features instead of the features of the generated images and the real images. Thus, we additionally employ a feature fusion-based loss function to train the generator, and the function is formulated as follows:


(5) $$L_{f\,u\,s\,i\,o\,n}\,\left(G\right)=E_{\text{y}∼P_{P\,E\,T},x∼P_{M\,R\,I}}\,\left[{||F\,\left(x,y\right)-F\,\left(x,G\,\left(x\right)\right)||}_{1}\right],$$

where F is a feature fusion model that is pretrained in section “Pretraining Classification Network.” The feature fusion model can extract and fuse data with different modalities and obtain the features that are most helpful for classification. The final loss function can be expressed as:


(6) $$\arg\,\min_{G}\max_{D}L_{G\,A\,N}\,\left(G,D\right)+\lambda_{1}\,L_{1}\,\left(G\right)+\lambda_{2}\,L_{f\,u\,s\,i\,o\,n}\,\left(G\right),$$

where λ_1_ and λ_2_ are hyperparameters.

The entire structure of our FF-GAN is found in Figure 1B. The GAN loss, estimation error loss and feature fusion-based loss are shown in Eqs (2), (3), and (5), respectively.

### Fine-Tuning Classification Network

Thus far, we have a classification model, but it is trained only by real MRI and PET images. There are errors between generated images and real images. Therefore, when we use the generated images with a certain error level for the early diagnosis of AD, the results may not be satisfactory. To solve these problems, we fine-tune the model using our FF-GAN, as shown in Figure 1C. Specifically, we assume that the PET scans in the training set are missing. We freeze the parameters of the generator trained in section “Feature Fusion-Based Generative Adversarial Network” and adopt it to synthesize PET images. Afterward, we use real MRI and synthesized PET images to fine-tune the previously trained classification network. Thus, the classification model can adapt to real data and generate data simultaneously.

### Network Architecture

**FIGURE 2:**
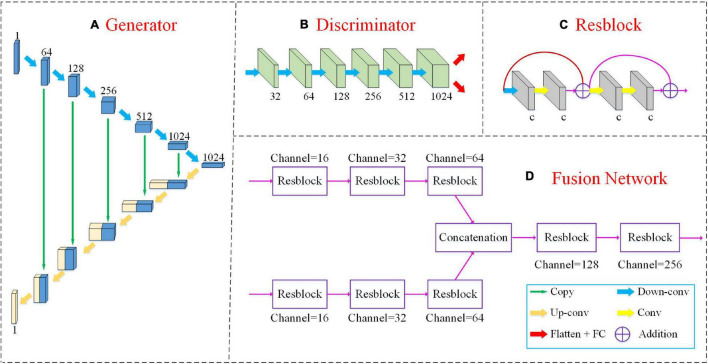
The detailed network structures of our method include a 3D U-Net generator, a discriminator and a multimodal fusion network as shown in panels **(A,B,D)**. The “Resblock” is the basic unit of the fusion network as shown in panel **(C)**. The green arrow “copy” implies that the two feature maps are connected along the channel dimension. “Down-conv” and “Up-conv” represent downconvolutional and upconvolutional operations, respectively. Both of them use 3 ×3 ×3 filters applied with a stride of 2. The yellow arrow “Conv” denotes a convolutional layer with 3 ×3 ×3 filters, but the stride is 1. The red arrow “Flatten+FC” indicates that we flatten the feature map into a vector and input it into the FC layer. “Addition” denotes the elementwise addition.

The detailed network structures of our method are shown in Figure 2, including a 3D U-Net generator, a discriminator and a multimodal fusion network.

Our FF-GAN is based on a traditional U-Net (Ronneberger et al., 2015). The generator consists of two sequential (i.e., encoding and decoding) parts. The encoding part is constructed by 6 upconvolutional layers with 64, 128, 256, 512, 1,024, and 1,024 channels. The decoding part consists of 6 downconvolutional layers with 1,024, 512, 256, 128, 64, and 1 channel(s). For the decoder part, we concatenate the feature maps obtained from the encoder and previous downconvolutional layers along the channel dimension and input the concatenated version into the current downconvolutional layer. In addition, the discriminator contains 6 convolution layers with 32, 64, 128, 256, 512, and 1,024 channels. For all convolution layers, 3 ×3 ×3 filters are used, and their strides are 2. The activation function is a leaky rectified linear unit (ReLU).

The fusion network is based on the residual network (ResNet) (He et al., 2016) model. We input MRI and PET images into different networks with the same structure to extract the features of different modalities. Next, we concatenate these two feature maps along the channel dimension. Finally, the connected feature maps are fed into the following networks to fuse the features of different modalities. The basic block of the fusion network is constructed by 4 convolution layers and 2 shortcut connections. All four convolution layers apply 3 ×3 ×3 filters, a ReLU activation function and batch normalization (Ioffe and Szegedy, 2015). The stride of the first convolution layer is set to 2, while those of the other three layers are set to 1. After each “Resblock,” we adopt dropout at a rate of 50% to avoid overfitting.

### Network Training

In stage 1, we pretrain the classification network with the entire MRI and PET images, each with a size of 128 ×128 ×128. Compared with the patch-based (Li and Liu, 2018), region of interest (ROI)-based (Aderghal et al., 2018) or slice-based (Liu et al., 2018b) methods, the use of the whole 3D images enables the model to better use the image information. The network is trained with the Adam solver (Kingma and Ba, 2014) with a learning rate of 0.0005. The batch size is set to 8. We also use the L2 regularization loss with a regularization term of 0.001 to avoid overfitting.

In stage 2, the pretrained fusion model of the classification network is used for GAN training. However, using the entire images is not effective for training the GAN. Therefore, we extract 3D patches from both MRI and PET images with sizes of 64 ×64 ×64 and step sizes of 32. Therefore, each image with a size of 128 ×128 ×128 can extract 27 patches, and all of these patches are used to train the GAN in a standard alternating manner (Goodfellow et al., 2020). Thus, we can simultaneously increase the number of training samples and significantly reduce the number of training parameters. Specifically, we first fix the generator network G and train the discriminator network D for 5 steps; then, we fix D and train G for 1 step. Both networks are trained with the Adam solver with a learning rate of 0.00001. Since we must calculate the feature fusion-based loss of Eq. (5), in each iteration, the 54 patches obtained from a pair of MRI and PET images are used for training. Both estimation error term λ_1_ and feature fusion-based loss term λ_2_ of Eq. (6) are set to 100.

In stage 3, we assume that the PET images are missing in the training set and adopt the generator to synthesize PET images. Afterward, real MRI and synthesized PET images are used to fine-tune the classification network. The learning rate is set to 0.0001, and the other parameters are identical to those in the pretraining stage.

## Experimental Setup

### Materials and Image Preprocessing

The Alzheimer’s Disease Neuroimaging Initiative (ADNI) dataset was set up in 2003. All participants across the ADNI-1, ADNI-2, and ADNI-GO studies signed informed consent forms. In our study, 360 MCI subjects, including EMCI and LMCI subjects, were downloaded from the ADNI dataset^1^. In our study, the slice thickness of most samples that we used was 1.2 mm. The field strength mainly includes 1.5 tesla and 3 tesla. All of them had MRI baseline data, but only 257 subjects had PET images. The paired dataset with all MRI and PET images included 124 EMCI and 133 LMCI patients, while the unpaired dataset with only MRI scans contained 27 EMCI and 76 LMCI patients. There were no significant differences between the two groups in gender, age, or education years. The detailed demographic information and clinical characteristics in this study are summarized in Table 1.

**TABLE 1:** Demographic and clinical characteristics of the subjects.

	Paired data			Unpaired data		
	EMCI (*n* = 124)	LMCI (*n* = 133)	*p*-value	EMCI (*n* = 27)	LMCI (*n* = 76)	*p*-value
Gender (M/F)	68/56	75/58	0.802	16/11	41/35	0.633
Age	70.1 ± 6.8	72.0 ± 7.4	0.358	72.1 ± 7.5	75.7 ± 7.1	0.614
Education	16.2 ± 2.6	16.1 ± 3.0	0.407	16.2 ± 2.4	15.2 ± 3.3	0.126
MMSE	28.1 ± 1.9	25.7 ± 3.8	<0.001	28.8 ± 1.1	26.8 ± 1.8	<0.001

*Age, education, and MMSE are shown as the mean ± standard deviation values. MMSE, mini-mental state examination; EMCI, early mild cognitive impairment; LMCI, late mild cognitive impairment; No significant diffierences were found between the two groups in gender, age, or education years. Groups for EMCI and LMCI showed significant diffierences in MMSE scores (p < 0.01). Statistical p-values were analyzed using a t-test, except for gender (chi-square test).*

We preprocessed all MRI and PET images by registration and skull stripping. We registered the MRI and PET images in three steps. (1) The PET images were mapped to their corresponding MR images; (2) the MR images were aligned to the Montreal Neurological Institute (MNI) space; (3) the PET images were mapped to the MNI space according to the deformation field produced in the second step. All registered images were checked by vision. Then, we segmented all images into gray matter, white matter, and cerebrospinal fluid and removed the skulls. Next, we removed the blank layers of all images and resized them to 128 ×128 ×128. Finally, we normalized the image intensity to [0,1] by the min-max normalization method.

### Evaluation Measures

To evaluate the classification and GAN performance of our method, we use seven measures in our experiments. The classification accuracy (ACC), sensitivity (SEN), specificity (SPE), and area under the receiver operating characteristic curve (AUC) were utilized to measure the classification performance. The mean squared error (MSE), peak signal-to-noise ratio (PSNR), and structural similarity (SSIM) were used to evaluate the performance of the GAN.

### Experiments and Data Partition

Our experiments are performed on incomplete and complete data, implemented in TensorFlow on NVIDIA RTX3090 GPU. Incomplete data experiments only use paired MRI-PET data to evaluate the effectiveness of our method. While complete data experiments use both paired and unpaired data to evaluate the effectiveness of the additional training data that are generated by GAN. For incomplete data experiments (from section “Performance of Classification With Single-Modality or Multimodal Data” to “Visualization of the Results of the Fused Features and the Most Critical Regions”), we divide the paired data into 10 subsets, nine of which form the training set, and the last is the validation set. Moreover, the unpaired data is treated as the testing set. For complete data experiments (section “Complete Data Experiments”), we redivide the paired data and unpaired data into 10 subsets. The training set includes nine of the subsets containing both paired data and unpaired data. The remaining is further divided into the validation set and testing set. To increase the amount of training data, data augmentation is conducted on the training set by horizontally and vertically flipping the images with a certain probability.

## Experimental Results

To evaluate the effectiveness of our method, the following six experiments were performed. (1) We compared the classification performance of single-modality data with that of multimodal data. (2) The performance of our FF-GAN was compared with that of the traditional GAN. (3) We compared the classification performance of our method with the multimodal result of experiment (1). (4) Our proposed method was further evaluated by an ablation study. (5) We visualized the results of the fused features and the most critical regions for EMCI and LMCI classification. (6) We synthesized the missing PET images of unpaired data and used all data to repeat experiments (3) and (4). Afterward, we compared our method with the state-of-the-art methods.

### Performance of Classification With Single-Modality or Multimodal Data

In this section, we evaluated the classification results of three networks. The first two are single-modality networks that are trained by all MRI or PET images, while the last one is a multimodal network that is trained by paired MRI and PET images. Specifically, the paired dataset is used for the multimodal network, and PET images from the paired dataset are used for the PET-based single-modality network. All MRI subjects from paired and unpaired datasets were used for the MRI-based single-modality network. For validation, the data are divided into 10 subsets, nine of which are the training set and the last is the validation set. The multimodal classification network is identical to that of subsequent experiments. It consists of a fully connected (FC) layer and a fusion network that can be found in Figure 2. The fusion network can be divided into three parts: two single-modality parts and a multimodal fusion part. We use the two single-modality parts with FC layers to train MRI and PET images, respectively. All three networks are trained with the whole 3D images, and the results can be found in Table 2. Table 2 shows that the accuracy obtained by the single-modality network with PET images is 1.66% better than that produced by MR images. The multimodal network has a higher accuracy than both single-modality networks with improvements of 5.51 and 3.85%, respectively. These results show that the use of PET images can achieve a better result than utilizing MRI, and applying multimodal data can distinctly improve the classification performance. Thus, it is necessary to use multimodal data for early AD detection, and our further experiments are all based on multimodal data.

**TABLE 2:** Classification performance of the single-modality and multimodal data on the validation set.

Input data	ACC (%)	SEN (%)	SPE (%)	AUC (%)
MRI only	67.57	66.67	68.42	71.93
PET only	69.23	**71.43**	66.67	73.21
Paired MRI and PET	**73.08**	**71.43**	**75.00**	**79.17**

*The bold values mean the best results.*

### Performance of Feature Fusion-Based Generative Adversarial Network Compared With That of the Traditional Generative Adversarial Network

To evaluate the effectiveness of our fusion model, we train the traditional GAN, p2pGAN, our FF-p2pGAN and FF-GAN with 3D patches of paired MRI and PET images. These patches are extracted from MRI and PET images with sizes of 64 ×64 ×64 and a step size of 32. The results of GANs are shown in Table 3, which shows that the synthetic images generated by our FF-GAN outperforms the traditional GAN in terms of the MSE, PSNR, and SSIM. Specifically, the MSE decreases by 0.00196, while the PSNR and SSIM increase by 0.47373 and 0.0069, respectively. Moreover, same conclusion is obtained for p2pGAN and FF-p2pGAN. These results show that the image generated by our FF-GAN is better than other GANs in terms of the global characteristics. To validate whether the synthesized images can improve the performance of disease, we conduct further experiments (from sections “Performance of Our Hybrid Deep Learning Method” to “Complete Data Experiments”).

**TABLE 3:** GAN results of a quantitative comparison.

Method	MSE	PSNR	SSIM
GAN	0.02004	23.44502	0.76464
FF-GAN	0.01808	23.91875	**0.77154**
p2pGAN	0.01936	23.76414	0.76244
FF-p2pGAN	**0.01763**	**23.93632**	0.76355

*The bold values mean the best results.*

### Performance of Our Hybrid Deep Learning Method

Based on the previous two experiments, we performed further research. Compared with the multimodal result in section “Performance of Classification With Single-Modality or Multimodal Data,” we assume here that the PET images of the training set are missing, and we use the trained FF-GAN from section “Performance of Feature Fusion-Based Generative Adversarial Network Compared With That of the Traditional Generative Adversarial Network” to synthesize PET images. Next, all real MRI and generated PET images in the training set are used to fine-tune the pretrained classification network of section “Performance of Classification With Single-Modality or Multimodal Data.” The results can be found in the upper part of Table 4. The validation set result of this table is obtained by inputting the real MRI and PET images of the validation set, while the testing set result is obtained from the real MRI and synthesized PET images of the testing set. The validation set result of GAN and FF-GAN is identical to that of section “Performance of Classification With Single-Modality or Multimodal Data.” Comparing the results of the validation set and testing set enables us to demonstrate the adaptability of the classification network with respect to the synthesized images. From this table, we can find that our method improves the accuracy by 3.84% on the validation set, but on the testing set, our method increases the accuracy by 5.82%. Thus, our method can better adapt to generated images and simultaneously improve the accuracy of real data. Moreover, we also conduct the above experiment by p2pGAN based on the same pretrained classification network of section “Performance of Classification With Single-Modality or Multimodal Data.” The results can be found in the lower part of Table 4 and we can get the same conclusion.

**TABLE 4:** Classification performance obtained using different methods.

Method	Validation set (%)				Testing set (%)			
	ACC	SEN	SPE	AUC	ACC	SEN	SPE	AUC
GAN	73.08	71.43	**75.00**	79.17	73.79	75.00	70.37	82.46
FF-GAN	73.08	71.43	**75.00**	79.17	77.67	76.32	81.48	85.04
GAN+fine-tune	**76.92**	**78.57**	**75.00**	83.93	77.67	76.32	81.48	84.60
Ours (FF-GAN+fine-tune)	**76.92**	**78.57**	**75.00**	**87.50**	**79.61**	**78.95**	81.48	85.19
p2pGAN	73.08	71.43	**75.00**	79.17	73.79	72.37	77.78	83.09
FF-p2pGAN	73.08	71.43	**75.00**	79.17	77.67	76.32	81.48	82.70
p2pGAN+fine-tune	**76.92**	**78.57**	**75.00**	86.31	77.67	**78.95**	74.07	**86.79**
Ours (FF-p2pGAN+fine-tune)	**76.92**	**78.57**	**75.00**	86.90	78.64	72.37	**96.30**	84.89

*The bold values mean the best results.*

### Ablation Study

In this section, we perform an ablation study by only using the feature fusion-based loss in the generator or by only fine-tuning the classification network. The results are shown in Table 4. The first and second rows use the multimodal classification network of section “Performance of Classification With Single-Modality or Multimodal Data.” However, the first row uses a traditional GAN for missing data generation, while the second row uses our FF-GAN. The third row also uses the traditional GAN for missing data generation and fine-tunes the classification network. The fourth row is our method, which uses our FF-GAN for image generation and fine-tunes the classification network. The last four rows of the Table 4 are achieved by p2pGAN. Comparing the first and second rows, we find that using the feature fusion-based loss can improve accuracy by 3.88% on the testing set. Comparing the first and third rows, we find that fine-tuning can improve the accuracies on both the validation set and testing set by 3.84 and 3.88%, respectively. Similarly, from the second and fourth rows, we find that the accuracies on both datasets increase by 3.84 and 1.94%, respectively. For p2pGAN, we can get the same conclusion. Therefore, both feature fusion-based loss of the GAN and the fine-tuning stage of the classification network are effective for improving the accuracy of the results. From Table 4, we can find that baseline GAN is slightly better than p2pGAN. Thus, we conduct further experiments based on baseline GAN instead of p2pGAN.

### Visualization of the Results of the Fused Features and the Most Critical Regions

**FIGURE 3:**
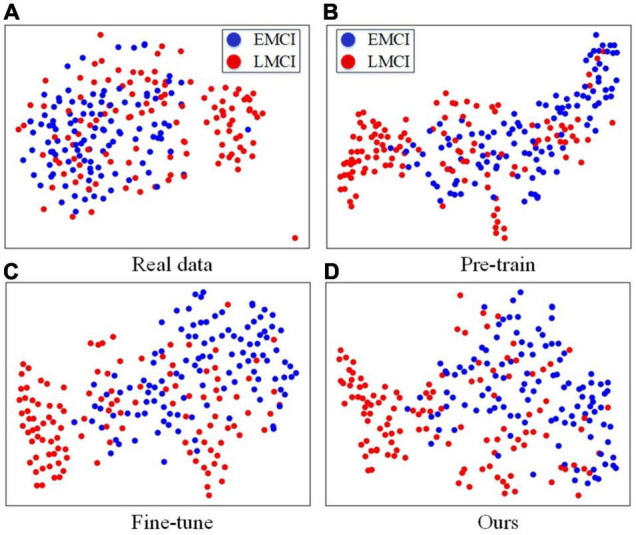
**(A)** t-SNE results obtained with real data. **(B–D)** t-SNE results obtained with the fused features of the pretrained classification model, fine-tuned model of the traditional GAN, and fine-tuned model produced by our FF-GAN.

In this part, we further analyze the fused features by visualization. The t-distributed stochastic neighbor embedding (t-SNE) (Maaten and Hinton, 2008) method is used to compare the classification difficulties of the compared methods, and the results can be found in Figure 3. For Figure 3A, we concatenate the MRI and PET images together, and we can easily find that it is difficult to separate the two clusters from the real data. Therefore, directly using real data for classification is more difficult than the other three methods. The t-SNE results obtained by using the fused features of the pretrained classification model, fine-tuned model of the traditional GAN, and fine-tuned model yielded by our FF-GAN are shown in Figures 3B–D, respectively. The results show that the EMCI and LMCI clusters of our method are more clearly separated than those of the other two methods. Thus, the fused features obtained by our method are more helpful for classification.

**FIGURE 4:**
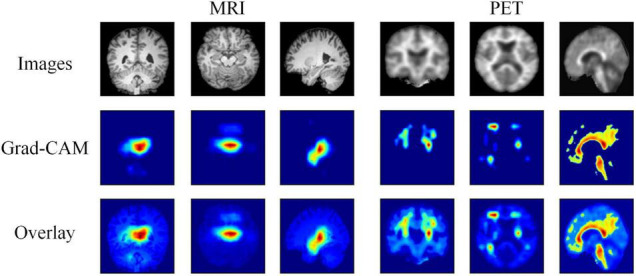
Visualization results of the MRI and PET images, the corresponding Grad-CAMs obtained by our method, and the associated overlays.

Moreover, we further visualize the gradient-weighted class activation mapping (Grad-CAM) (Selvaraju et al., 2017) of our hybrid deep learning method, as shown in Figure 4. This reflects which regions are most critical for EMCI and LMCI classification. We can clearly find that our method easily covers the hippocampus and temporal lobe of each MRI image and the cingulate gyrus and precuneus of each PET image. These regions have been related to the progression of AD.

### Complete Data Experiments

In this section, we first synthesize the missing PET images of unpaired data using the traditional GAN and our FF-GAN. Afterward, we redivide the paired data and unpaired data into 10 subsets. Nine of the subsets containing both paired data and unpaired data are training sets. The remaining paired data and unpaired data constitute the validation set and testing set, respectively. Finally, the experiments of sections “Performance of Our Hybrid Deep Learning Method” and “Ablation Study” are repeated by fivefold cross validation, and the results are shown in Table 5.

**TABLE 5:** Classification performance in complete data experiments by fivefold cross validation.

Method	Validation set (%)				Testing set (%)			
	ACC	SEN	SPE	AUC	ACC	SEN	SPE	AUC
GAN	76.65	76.69	76.61	85.32	73.79	75.00	70.37	75.97
FF-GAN	81.71	78.19	85.48	89.02	82.52	**85.53**	74.07	88.99
GAN+fine-tune	83.27	78.95	87.90	90.75	80.58	82.89	74.07	88.00
Ours (FF-GAN+fine-tune)	**85.21**	**79.70**	**91.13**	**93.64**	**84.47**	**85.53**	**81.48**	**92.81**

*The bold values mean the best results.*

From the first and last rows, we find that our method increases the accuracies on the validation set and testing set by 8.56 and 10.68%, respectively. This result indicates that the images generated by traditional methods are not good for classification. Moreover, our method can significantly improve the quality of the generated images and improve them for the classification task. From the first and second rows, we find that using only the feature fusion-based loss can improve the accuracies by 5.06 and 8.73%, respectively. This result shows that images generated by the FF-GAN are good for classification. Moreover, the use of the FF-GAN to synthesize the missing PET images of unpaired images can improve the pretraining results. Comparing the first and third rows, we find that fine-tuning can improve the accuracies by 6.62 and 6.79%, respectively. Similarly, from the second and last rows, we find that the accuracies obtained on the validation set and testing set are increased by 3.5 and 1.95%, respectively. These results show that the fine-tuning stage can obviously improve the accuracy on the testing test without losing or even improving the accuracy on the validation set. Additionally, comparing Table 4 with Table 5, we find that our method increases the accuracies by 8.29 and 4.86% for the validation set and testing set, respectively. Similarly, most of the results of Table 5 are much higher than those in Table 4. These results show that synthesizing missing data can improve the classification performance, especially when using our proposed FF-GAN. Finally, we compare our method with the state-of-the-art methods, as shown in Table 6. The comparison illustrates that our method can boost the classification performance between EMCI and LMCI on a relatively large dataset, which makes the results more reliable.

**TABLE 6:** Result comparison of other EMCI vs. LMCI classification methods.

Algorithm	Subjects	ACC (%)	SEN (%)	SPE (%)	AUC (%)
Jie et al. (2018)	56EMCI+43LMCI	78.80	74.40	82.10	78.30
Nozadi and Kadoury (2018)	164EMCI+189LMCI	72.50	79.20	69.90	79.00
Wee et al. (2019)	899EMCI+638LMCI	60.90	52.50	67.80	N/A
Lei et al. (2020)	44EMCI+38LMCI	81.71	78.95	84.09	**92.11**
Yang et al. (2021)	29EMCI+18LMCI	80.85	N/A	N/A	84.87
Ours	151EMCI+209LMCI	**83.78**	**80.95**	**87.50**	88.99

*The bold values mean the best results.*

## Discussion

In the present study, we used the hybrid deep learning method to classify patients with EMCI and LMCI, and the results show that our method improves classification performance. Here, we interpret the reasons. Traditional methods divide generation and classification into two parts with no information communication. However, in our proposed method, the pretrained classification network is used to obtain crucial features for GAN training, and the synthesized images are used to fine-tune the classification network. Additionally, we further strengthen the information communication between two networks by iterating the two stages. Thus, the resulting GAN can focus on the crucial regions that are helpful for classification. Moreover, we transfer the model trained by real images to synthesized images by fine-tuning stage, which may make the classifier better adapt to the generated image.

There are also several studies to make the generated images suitable for classification. Gao et al. (2021) propose a task-induced discriminator to include the abnormal changes of generated medical images. However, there is no information communication between the classification network and task-induced discriminator. Pan et al. (2019, 2021) propose a disease-image-specific deep learning framework for joint neuroimage synthesis and disease diagnosis, which realize the information communication between the two networks. But this method extracts disease-image specific information of MRI and PET images, separately. Thus, the fusion information of different modalities is not fully considered. In our method, the proposed FF-GAN focus more on fusion information and the multimodality network is utilized to promote its training, which can strengthen the information communication between two networks.

The ablation study shows that both feature fusion-based loss of the GAN and fine-tuning stage of the classification network are effective for improving the accuracy of the results. The former can extract regions that are helpful for classification, which helps the GAN better generate images. The latter can make the classifier better adapt to the synthetic image, which is particularly important when using incomplete datasets. Moreover, the visualization of the results shows that the fusion features extracted by our method can better realize the classification of EMCI and LMCI, and our method can easily cover key areas that are helpful for classification.

Complete data experiments indicate that using more data by synthesizing missing PET images can improve the classification performance. Using images generated by our FF-GAN is especially good for classification with the help of the feature fusion-based loss. In addition, we compare our method with the state-of-the-art methods for classifying EMCI and LMCI. The results show that our method achieves better classification performance than all listed algorithms on a large dataset, which makes the results more reliable.

However, we acknowledge that our study has two limitations. (1) The image preprocessing stage used for this study is handcrafted. This process depends on the experience of experimenters and software. Therefore, it can hardly be optimized for other datasets. (2) We use only the paired ADNI dataset for training. However, adding more data can improve the classification performance of the resulting model. Therefore, in our future work, we will combine other datasets with the ADNI dataset.

## Conclusion

In this paper, we propose a hybrid deep learning method for early AD detection with incomplete multimodal data. Our method has three stages. Specifically, in the first stage, we use a paired dataset to pretrain the classification network. In the second stage, we introduce a feature fusion-based loss into the traditional GAN using the pretrained classification network. Thus, the generator can better focus on the regions that are helpful for classification. In the last stage, we use real MR images and the generated PET images to fine-tune the classification model to better adapt to the synthesized images. The results show that our method is superior to the state-of-the-art methods for early AD detection and can better handle incomplete multimodal datasets. Moreover, the feature fusion-based loss and fine-tuning stage are complementary for early AD detection. Additionally, applying an entire dataset that is synthesized by our method can significantly improve the resulting classification performance.

## Data Availability Statement

The original contributions presented in the study are included in the article/supplementary material, further inquiries can be directed to the corresponding author/s.

## Ethics Statement

The studies involving human participants were reviewed and approved by Alzheimer’s Disease Neuroimaging Initiative (ADNI). The patients/participants provided their written informed consent to participate in this study. Written informed consent was obtained from the individual(s) for the publication of any potentially identifiable images or data included in this article.

## Author Contributions

LJ contributed to conceptualization, methodology, software, validation, writing—original draft, and visualization. KZ contributed to investigation, resources, and visualization. YZ and TC contributed to writing—review and editing. SL contributed to supervision, project administration, and funding acquisition. All authors contributed to manuscript revision, read, and approved the submitted version.

## Conflict of Interest

The authors declare that the research was conducted in the absence of any commercial or financial relationships that could be construed as a potential conflict of interest.

## Publisher’s Note

All claims expressed in this article are solely those of the authors and do not necessarily represent those of their affiliated organizations, or those of the publisher, the editors and the reviewers. Any product that may be evaluated in this article, or claim that may be made by its manufacturer, is not guaranteed or endorsed by the publisher.

## Footnotes

1
adni.loni.usc.edu
